# Surface Cleaning of SAW-Based Microparticle Sensors Integrated in a Cascade Impactor Using SAW-Induced Droplet Displacement †

**DOI:** 10.3390/s26154666

**Published:** 2026-07-23

**Authors:** Ghida Fawaz, Meddy Vanotti, Sacha Poisson, Hiba Taleb, Virginie Blondeau-Patissier

**Affiliations:** Université Marie et Louis Pasteur, CNRS, Institut FEMTO-ST (UMR 6174), F-25000 Besançon, France; meddy.vanotti@femto-st.fr (M.V.); sacha.poisson@femto-st.fr (S.P.); hiba.taleb@femto-st.fr (H.T.)

**Keywords:** acoustic streaming, cascade impactor, droplet displacement, lithium niobate, particle sensors, particulate matter, Rayleigh waves, surface acoustic waves

## Abstract

A cascade impactor equipped with microparticle surface acoustic wave sensors along with a surface cleaning system is an innovative system developed by our team to measure particles and monitor air quality. The system has been proven to function properly under various particle concentrations. Nevertheless, long exposure times and heavily polluted media impose a limitation on cascade impactors, known as surface saturation. This problem affects the sensitivity of our sensors, which tends to degrade with particles fouling the surface. To overcome this issue, a surface cleaning system that uses a Rayleigh wave-actuated water droplet has been implemented and tested. Rayleigh waves were generated on an innovative SAW chip, thus exciting the droplet using Radio-Frequency power. NaCl solutions and SiC particles were considered. The optimal droplet size was determined along with the required Radio-Frequency power. Experiments showed that the SAW-driven droplet successfully displaced the collected particles outside of the sensing zone, irrespective of their nature, without affecting the sensor’s performance. The results found in this study provide further improvements to our particle measuring system, advancing it towards an autonomous self-regenerating prototype.

## 1. Introduction

The topic of air pollution has been attracting international interest. Particulate matter (PM), which constitutes particles of various sizes and origins, is one of the main causes of this issue. The particles’ small size allows them to penetrate deeply into our respiratory system, thus causing severe and chronic respiratory and cardiovascular diseases, as well as high rates of premature deaths. Air quality monitoring has become an essential need to avoid these consequences. Several devices existing on the market tackle this issue and monitor PM concentrations. Optical Particle Counters (OPCs), for instance, are one way to measure PM [1,2]. However, they are sensitive to particles’ shape and nature since they rely on light diffraction as a result of light beam–particle interactions. Gravimetric methods are, hence, preferred. Several gravimetric devices are also available on the market [3,4,5,6,7]. These will not be discussed further, as they are outside the scope of this paper. However, one particular inconvenience is highlighted among them, and that is the constant need for maintenance. In line with these needs, and over the past decade, an original particle measurement system was developed and patented by our team (FR3050031A1) and is presented in detail in the work of Djoumi et al. [8,9]. Since then, we have worked on the optimization of the entire system. The original system consisted of a cascade impactor comprising three stages, two of which were equipped with Surface Acoustic Wave (SAW) sensors to measure the collected particles. This device not only guaranteed particle separation based on particle size, but it also enabled measuring them. The impactor used to have threaded rods and bolts as the assembly mechanism, introducing a risk of sensor damage.

**Figure 1:**
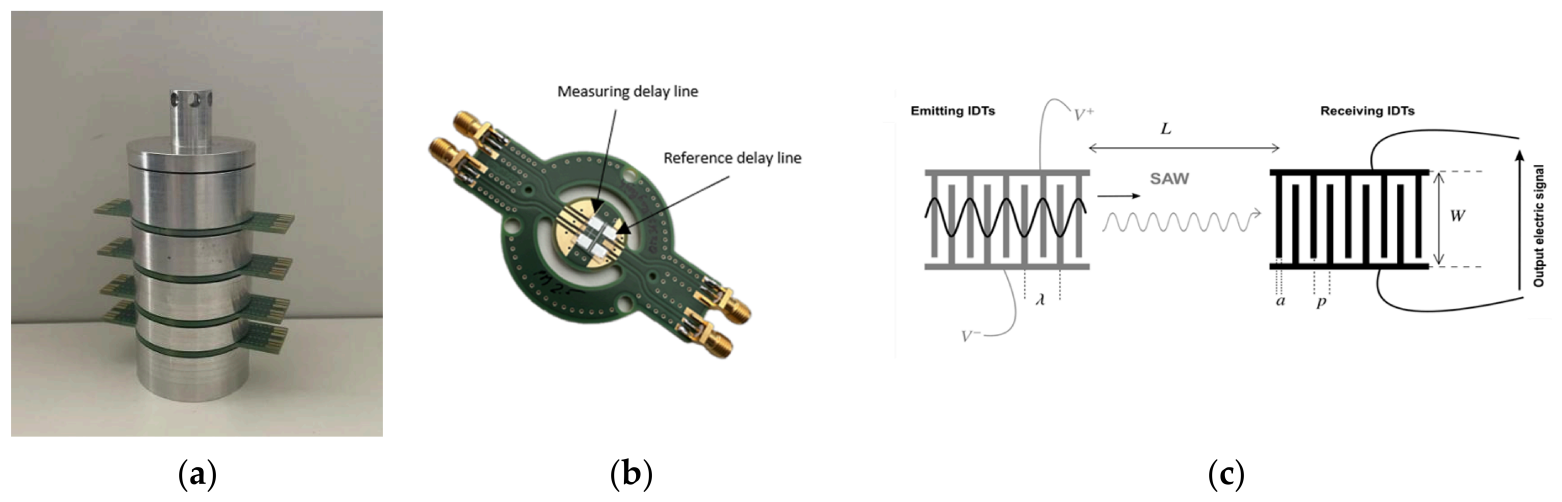
Image (**a**) shows the new cascade impactor with four stages to filter coarse particles and measure PM10*, PM2.5* and PM1, while image (**b**) displays the SAW sensors mounted on a PCB that matches the new design of the impactor with one measurement delay line and another reference delay line; and image (**c**) shows the delay-line configuration with two sets of IDTs, one acting as an emitter and the second one as a receiver [9].

In the new prototype, an additional stage was designed and added to measure PM1 (less than 1 µm). An ergonomic design incorporating a magnetic assembly was implemented to simplify handling, ensure accurate alignment of the stages while guaranteeing a homogeneous pressure distribution on the joints between the stages, and secure a leak-proof stack, as seen in Figure 1. The new prototype now consists of a four-stage cascade impactor, instead of three stages. The first stage filters out coarse particles, like the original one. The other three stages are used to collect a specific size range of particles owing to the stage design, which takes into account the adequate cut-off diameter. The quantification of PM is possible due to the use of delay-line sensors based on SAWs using piezoelectric substrates to perform real-time PM mass concentration measurements in each of the three stages, mainly PM1, PM2.5* and PM10*, with an aerodynamic diameter less than 1.0 µm, between 1.0 and 2.5 µm, and between 2.5 and 10 µm, respectively. These sensors follow the original sensor design, having a delay-line configuration and consisting of two sets of interdigital transducers (IDTs): the first set emits SAWs by applying an electric signal and the second set receives it as a mechanical output, which is then translated to an electric response. Another identical delay line is placed in parallel to the first one and is taken as a reference, as was the case in the original device. These two sensors are then mounted on a Printed Circuit Board (PCB). The delay-line working principle relies on measuring the variation in the wave’s phase velocity when facing a condition change on the surface, in this case, mass loading. The final output of the sensors is a differential measurement between the measurement line and the reference line to remove the influence of outer parameters such as humidity. 

To experiment with these sensors, the PCBs equipped with SAW sensors are integrated as impaction plates in the cascade impactor. The prototype is then placed in a leak-proof chamber in which a particle generator using NaCl and SiC solutions is set to generate particles of different sizes. A 3 Lpm pump connected to the output of the impactor is then used to collect aerosol samples. On their way through the impactor stages, the particles are separated and collected on the sensors.

**Figure 2:**
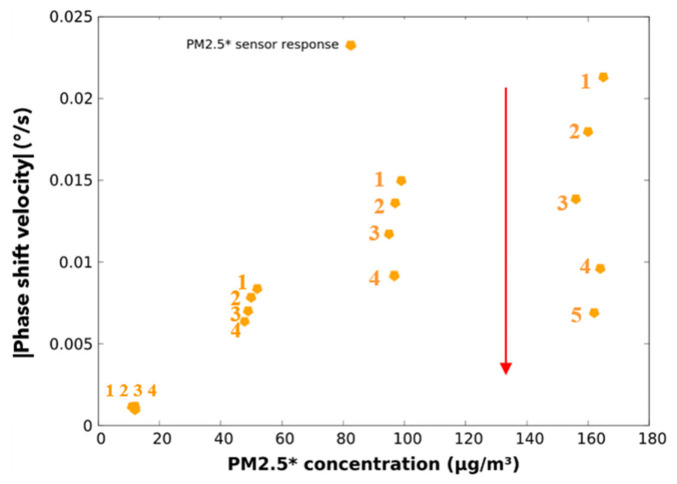
Monitoring of the PSV with respect to PM2.5* concentrations during a two-minute sampling per measurement. The numbers correspond to the order of the successive samplings carried out. This enables the visualization of the effect of surface saturation on the sensor’s performance, aligning with the temporal direction of the red arrow [11].

During the process of testing our system, the experiments aimed at evaluating the repeatability criterion of our sensors’ measurements prompted further investigation. We recorded four to five samplings at constant PM2.5* concentrations ranging between 10 and 170 µg/m^3^. Figure 2 presents the response obtained under different concentrations using the new impactor [10,11]. For every concentration tested, a new sensor was used, and the two-minute samplings were recorded consecutively using this same sensor. These results showed that, at low PM concentrations, the sensors’ readings, represented by the Phase Shift Velocity (PSV), are repeatable. However, at concentrations higher than 40 µg/m^3^, we noticed that the response decreased with each successive sampling. These results show a reduction in sensor sensitivity due to the accumulation of particles, necessitating cleaning prior to reuse. This observation underscores the effect of surface saturation on sensor performance and sensitivity.

**Figure 3:**
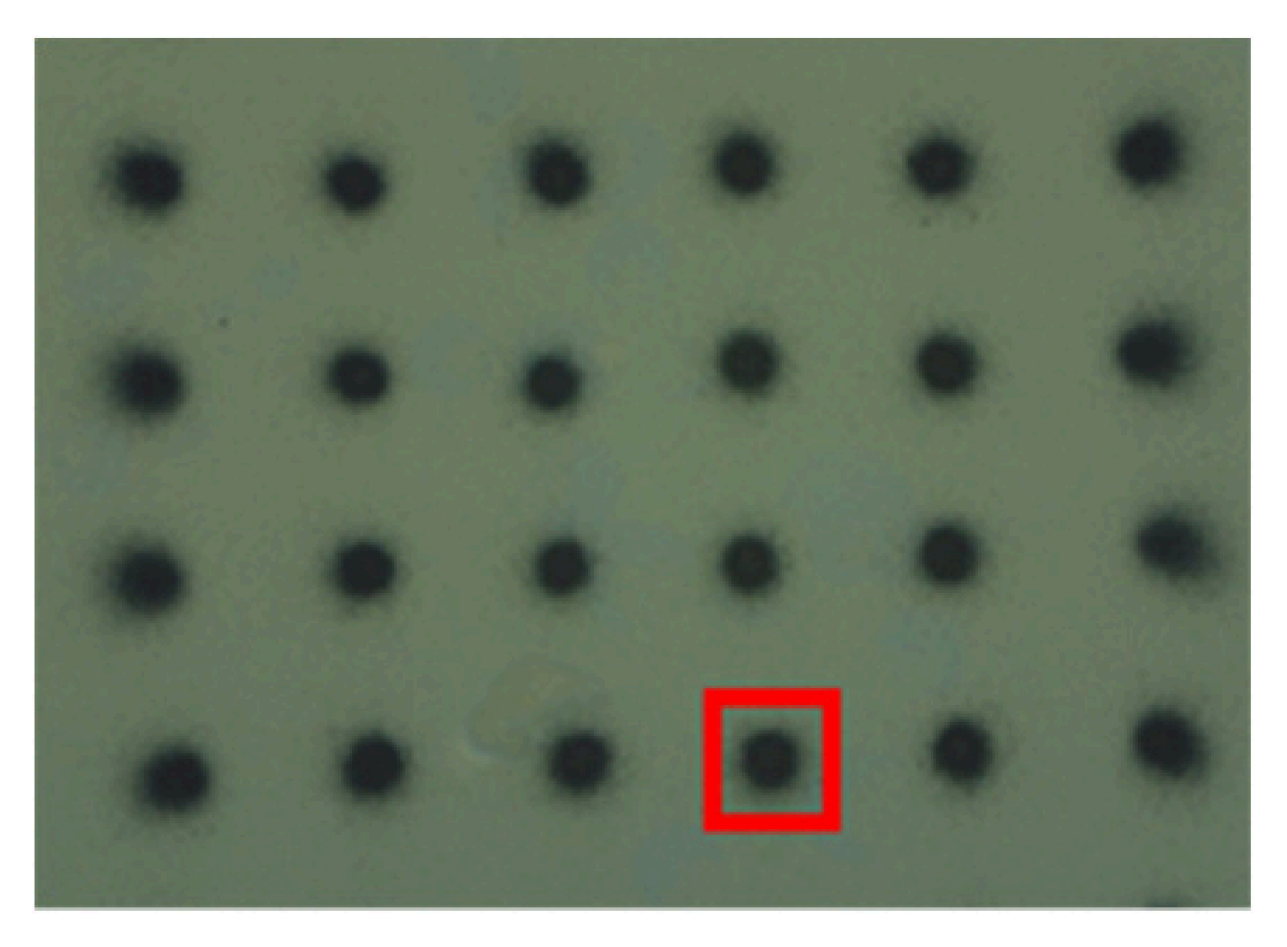
Top view of a PM1 sensor showing the collection surface and the spots where the particles were accumulated during the 20-min-long experiment under 330 µg/m^3^. The collection surface has an area of 0.1114 cm^2^ and the spots have an average diameter of 225 ± 7 µm. The red frame represents one of the spots.

**Figure 4:**
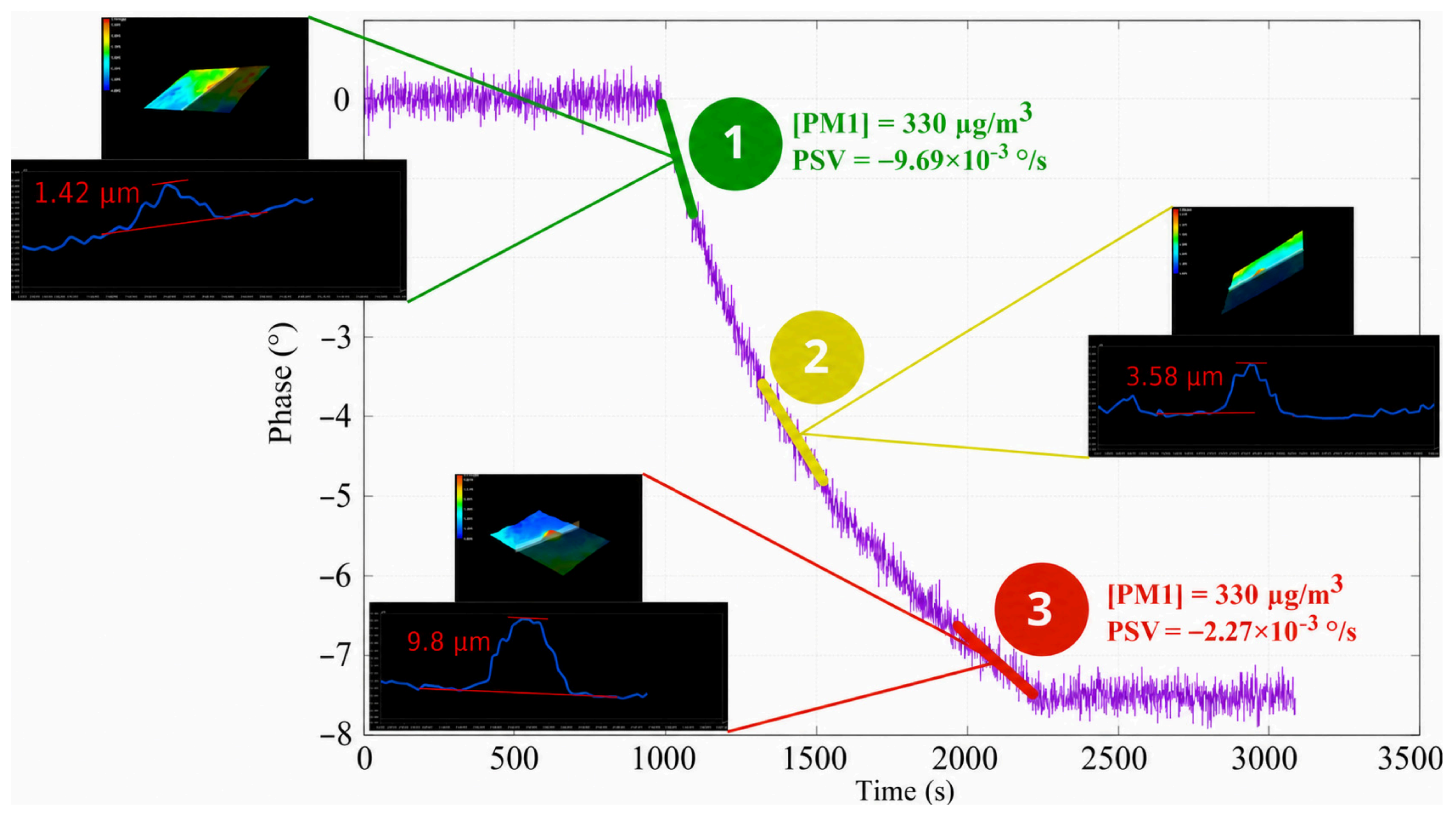
Three-dimensional characterization of single spots of particles seen on the surface of the PM1 sensor obtained under the Keyence microscope, captured at the beginning, in the middle, and at the end of the 20-min sampling experiment. At each time interval, the thickness of the particle accumulation was determined. The PSVs recorded by the PM1 sensor at a constant PM1 concentration of 330 µg/m^3^ were also noted for phase 1 and 3 to highlight the drop in the sensitivity.

To further endorse this premise, and based on the observations of the previous experiments, additional tests were conducted to investigate the surface saturation phenomenon from another perspective, using the same impactor, sensor design, and particle type. Hence, PM1 was considered, as this problem is more detrimental at this stage, as seen in Figure 3. In Figure 4, the response of a blank PM1 sensor is recorded under a highly polluted environment with a PM1 concentration of 330 µg/m^3^. The sensor’s phase was monitored during a continuous sampling of tens of minutes. In the first five minutes, the profile of the phase shift in time was linear. This is designated as phase 1, during which the sensor was operating in optimal working conditions. As the sampling advanced and particles continued to accumulate on the sensor, this linearity started to deviate, signaling a decline in the sensor’s sensitivity. This trend persisted for approximately 20 min, during which the sensor’s sensitivity continued to decrease, reaching phase 3.

To provide greater insight into the observed behavior, optical characterization was carried out to further explain this phenomenon. Three-dimensional images were constructed to observe the shape of particle clusters on the spots inside the sensing zone using a numerical microscope at three different intervals: a couple of minutes, 10 min, and 20 min from the start of the experiment. As seen in Figure 4, phase 1 exhibited an accumulation of approximately 1.5 µm, whereas a thickness of 3.6 µm was reached after 10 min in phase 2. The thickness then spiked in phase 3 to 9.8 µm after about 20 min. The increase in the buildup thickness was directly linked to the continuous loss of response linearity, as evidenced by the observed decrease in the PSV values from phase 1 to phase 3. Therefore, it was concluded that surface saturation was the underlying phenomenon responsible for the deterioration of sensor sensitivity and that this is likely attributed to the loss of interaction between the wave and the accumulated particles. These results emphasize the need to restore the sensor surface to its initial condition to ensure reliable and reproducible performance.

The behavior seen for the PM2.5* and PM1 sensors highlights the necessity of incorporating a cleaning mechanism into our system in order to ensure the continuous use of the sensors and extend their service life without the need to constantly replace them. In the meantime, there does not exist any gravimetric particle measurement system that includes an autonomous filter replacement or cleaning feature, as they all require the intervention of an operator. For this reason, the objective of this study was to upgrade our prototype by developing a surface-cleaning system capable of maintaining long-term operational durability of the sensors with minimal human intervention and without requiring dismounting the impactor to access the active sensing surfaces.

Since our sensors are based on SAWs, we proposed exploiting these waves to implement an efficient self-cleaning mechanism. The concept relies on using SAWs to induce the displacement of a water droplet deposited on the saturated region of the sensor, namely the particle-collection surface. As the droplet traverses the surface, it entrains and removes the accumulated particles from the active sensing area. Building on this, the present work investigates the feasibility of employing SAW-driven droplet actuation for particle removal from the sensor surface. To this end, we introduce a novel SAW chip precisely designed for this cleaning application. The study provides an optical demonstration of the cleaning process and evaluates its impact on sensor performance and measurement. This article is a revised and expanded version of a paper entitled Surface Cleaning of Microparticles Sensors Integrated in a Cascade Impactor via Water Droplet Displacement Using Surface Acoustic Waves, which was presented at the 2025 Joint Conference of the European Frequency and Time Forum and IEEE International Frequency Control Symposium held in Mexico in 2025 [11].

## 2. Materials and Methods

The proposed surface-cleaning mechanism calls for the design of a new SAW chip incorporating an additional IDT dedicated to cleaning. This section presents the new chip, along with a brief description of the physical phenomena governing droplet displacement induced by SAWs. The experimental setups used for the evaluation are also described.

### 2.1. SAW Sensor Configuration and Signal Processing

Prior to presenting our experimental methods, it is imperative to concretely describe how our sensors work in order to comprehend the analysis of the results. To recall, our SAW sensors are based on two delay lines used in a differential configuration. They are based on lithium niobate that excites Rayleigh waves at 98 MHz with an electromechanical coupling coefficient *k*^2^ of 5.5%. The interdigital transducers present a split-finger configuration to limit internal reflections. Conventional photolithography procedures were used to fabricate the 200 nm-thick aluminum electrodes. The mechanical period *p* is 10 µm; hence, the wavelength *λ* is 40 µm and the acoustic opening *W* is equal to 87*λ*.

**Figure 5:**
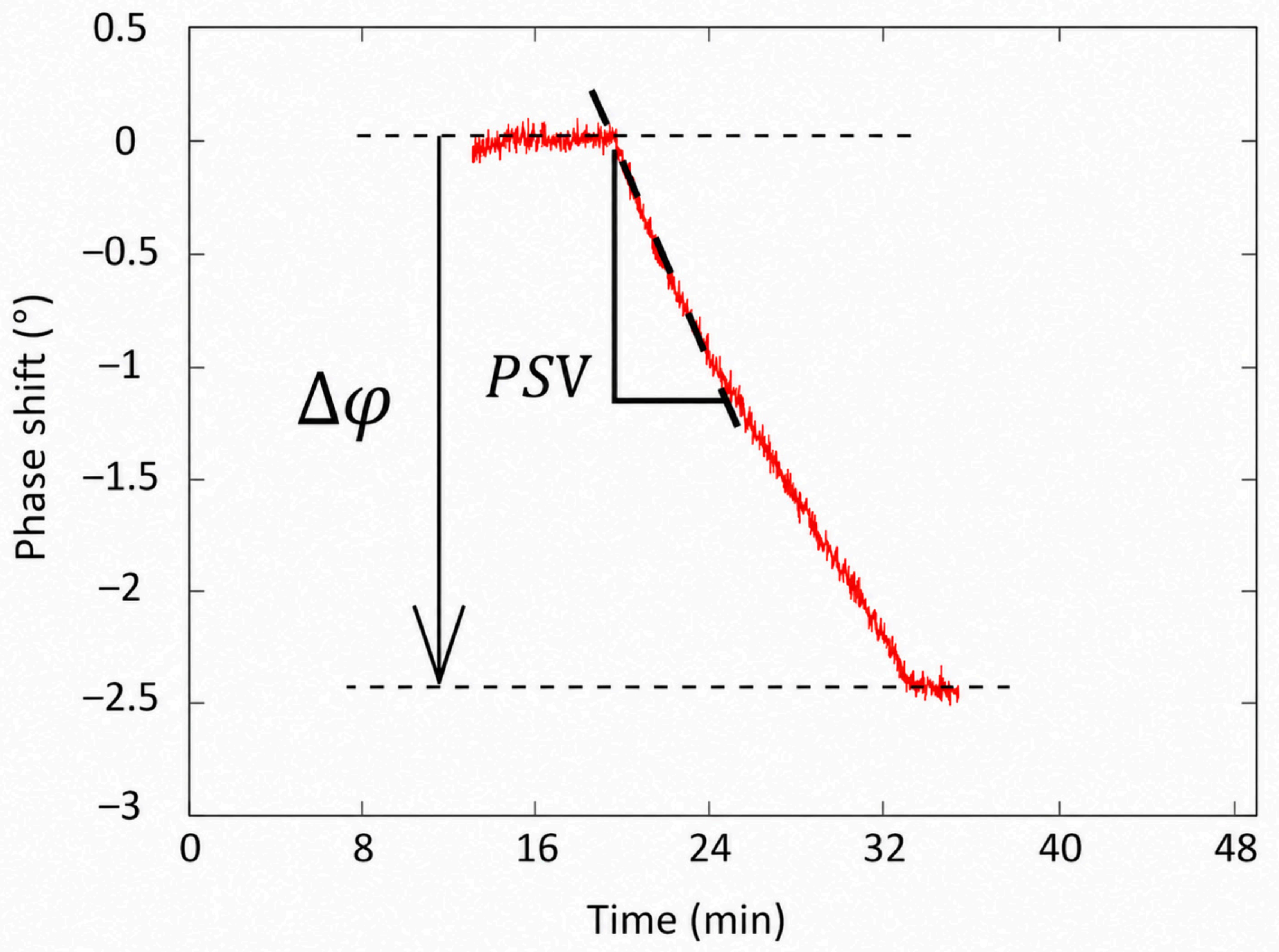
A graph showing the output of our delay-line sensor giving the phase shift upon mass deposition in time. The phase derivative in time is the PSV. This response was obtained over a 10-min sampling period.

As particles accumulate on the sensing zone, a mass loading effect is induced. This is shown in Figure 5, displaying an example of the sensor’s output [10]. It shows the phase shift upon particle deposition in time. The slope is associated with particle accumulation under a certain PM concentration and represents what we define here as the Phase Shift Velocity (PSV). To recall, the PM concentrations are recorded by an OPC, taken as our reference. The relationship between the deposited mass and the phase shift measured by the delay line can actually be explained by Sauerbrey’s equation of gravimetric sensitivity. This equation relates the change in frequency to the change in mass. As discussed, the effect of mass on the sensors is represented by a negative phase shift correlated with the particle concentration in the aerosol. This correlation has been previously established and detailed in the work of Djoumi et al. [8,9]. It begins with the gravimetric sensitivity *S* of SAW devices given by Sauerbrey’s equation [12]:

(1) $$S=\frac{df}{f_{0}}.\frac{A}{dm}$$

with *A* being the sensing surface and *f*_0_ being the working frequency. These two parameters are constants; any variation in the mass *dm* leads directly to a decrease in the frequency *df*. It is also important to emphasize that a linear relationship exists between the frequency and phase of SAW devices around their synchronous frequency. From this equation, and taking into consideration the parameters related to the impactor system, which are the flowrate and the sampling time, as well as the particle concentrations *C* given by the OPC, the following relationship was deduced:

(2) $$C\propto \frac{d\Phi }{dt}=PSV$$

Hence, it can be concluded that the particle concentration *C* is directly proportional to the phase variation over time $\frac{d\Phi }{dt}$, which is obtained from the sensors’ measurements and is designated by the *PSV*.

### 2.2. SAW-Induced Droplet Displacement for Surface Cleaning

**Figure 6:**
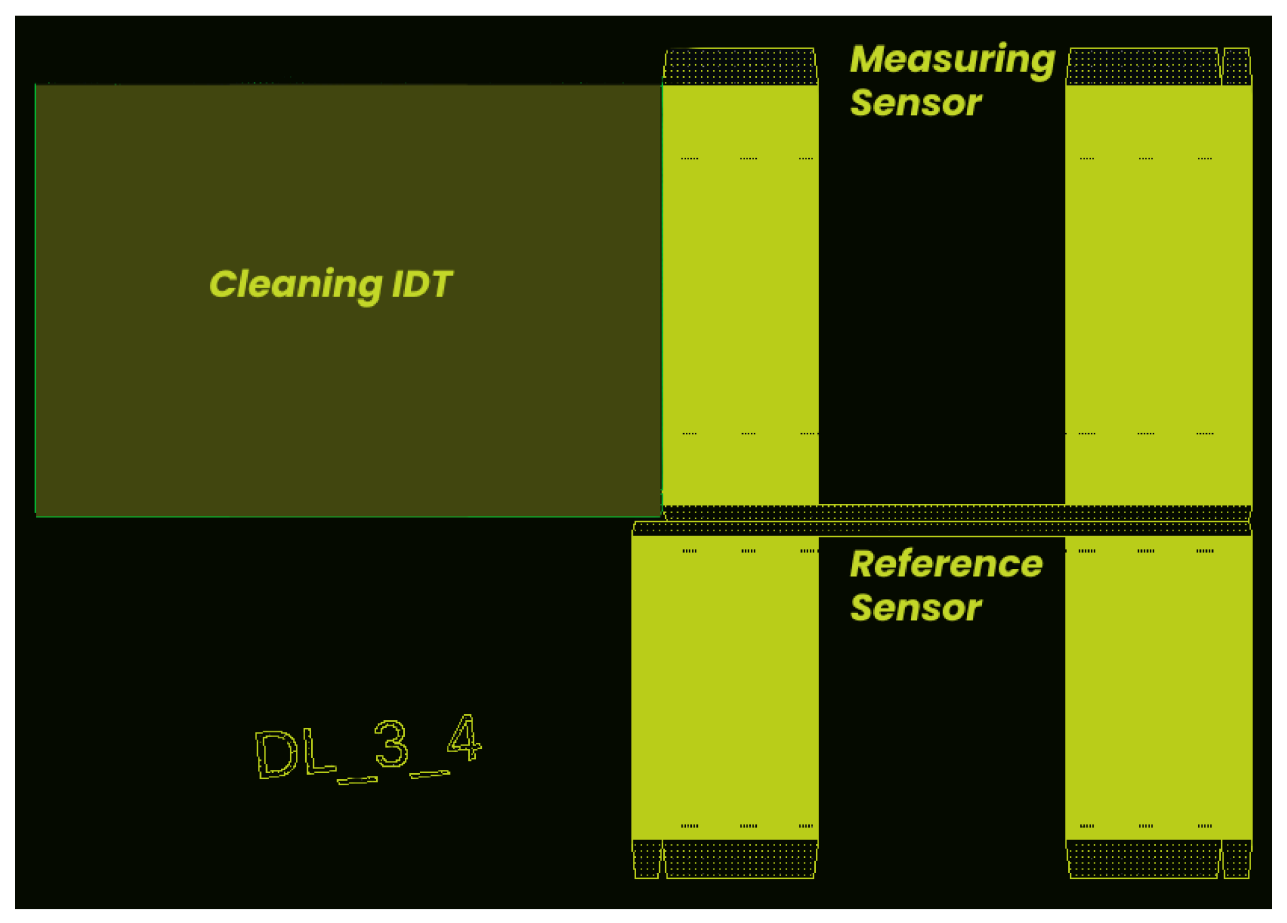
A representation of the new SAW chip. It shows the measurement sensor and the reference sensor, both operating at 98 MHz. The new cleaning IDT, operating at a different frequency, was mounted next to the measurement delay line.

As stated in the introduction, the exploitation of SAWs for the surface-cleaning mechanism was considered. In this context, the novelty of our device lies in the design of its chip, which includes an additional cleaning IDT placed alongside the measurement delay line and aligned on the same axis, enabling SAW-driven droplet displacement for sensor cleaning. Consequently, the proposed SAW chip performs a dual function, combining particle measurement and surface cleaning. The cleaning IDT operates at a different frequency from that of the measurement delay line. Due to the innovative nature of its design, the orientation and detailed specifications of the cleaning IDT are not disclosed for confidentiality reasons; however, it is important to note that it operates at a frequency of around 20 MHz and features a larger acoustic aperture than the measurement IDTs. A schematic representation of the new chip is presented in Figure 6.

Displacing droplets with SAWs has already been documented in various studies. SAWs, notably Rayleigh waves, have been exploited in numerous applications: in the development of an atomizer for medical applications by Kurosawa et al. [13], in lab-on-chip devices to run laboratory tests to help in the diagnosis of diseases [14], in DNA matching tests and hybridization via fluids stirring and mixing [15], and in high-precision applications where the droplet is manipulated with a 10 µm precision by Alzuaga et al. [16]. Besides these applications, the use of SAWs to displace a droplet for surface-cleaning purposes has also attracted the attention of researchers. This has been reported in the research field of Brunet et al. using lithium niobate-based SAW sensors as well as in the thesis work of Paquit and Alzuaga [17,18,19]. The motion of the droplet has been documented and filmed with high-velocity cameras, revealing some creeping and jumping in the device designed by Brunet et al. [17]. The design aimed to remove droplets from the surface, an interesting application, especially in the automotive industry, where sensors need to be regularly cleaned. This is also addressed in the surface-cleaning experiment conducted by Song et al., where SAW actuators were able to move a 2 µL water droplet over a distance of 16 mm [20].

The principle of SAW-induced droplet displacement is complex, as two main physical phenomena are simultaneously involved: acoustic streaming and radiation pressure. The substrate used in most of these applications is lithium niobate. Rayleigh waves are excited with an electromechanical coupling coefficient of 5.5% and propagate with elliptical polarization, provoking deformation of the surface owing to its out-of-plane component [21]. This is important, since an acoustic amplitude of a few nanometers is needed to induce a disturbance in a droplet of a few microliters, as this amplitude is directly related to the *k*^2^ [22]. For instance, waves with an amplitude of 2 nm can effectively displace droplets, whereas higher amplitudes exceeding 10 nm can atomize them [23].

To promote the displacement of the droplet, a system using Radio-Frequency (RF) pulsed excitation is required. The droplet behaves depending on the input power and, consequently, according to the phenomenon being involved. At low power, Rayleigh waves at low frequencies (~20 MHz) can agitate the water droplet and promote its oscillation at frequencies less than 150 Hz. These oscillations together with the wave’s action on the droplet elicit non-linear acoustic forces responsible for displacing the droplet with the onset of internal circulations. The first phenomenon detected is the internal flow of the fluid, designated by “acoustic streaming,” and was originally described by Rayleigh in 1884 [24]. As the RF power increases, acoustic radiation pressure arises [21]. Due to the fact that droplet motion relies on its ability to absorb energy from the incident wave, it is crucial to highlight the importance of droplet size. The droplet in question is of a few microliters, permitting us to disregard the effects of inertia on its motion, leaving surface tension as the main force acting on it. For each sensor design and operating frequency, careful optimization of droplet size is required to ensure efficient SAW-induced droplet actuation while preventing acoustic energy dissipation into the substrate. The droplet volume must be large enough to absorb the acoustic energy of the propagating wave, yet sufficiently small to remain below the capillary length (approximately 2.5 mm for water at room temperature), a parameter determined by surface tension. In this regime, inertial effects on droplet motion are negligible and may, therefore, be disregarded. Otherwise, the inertia could induce non-linear effects and the droplet may struggle to move [17].

**Figure 7:**
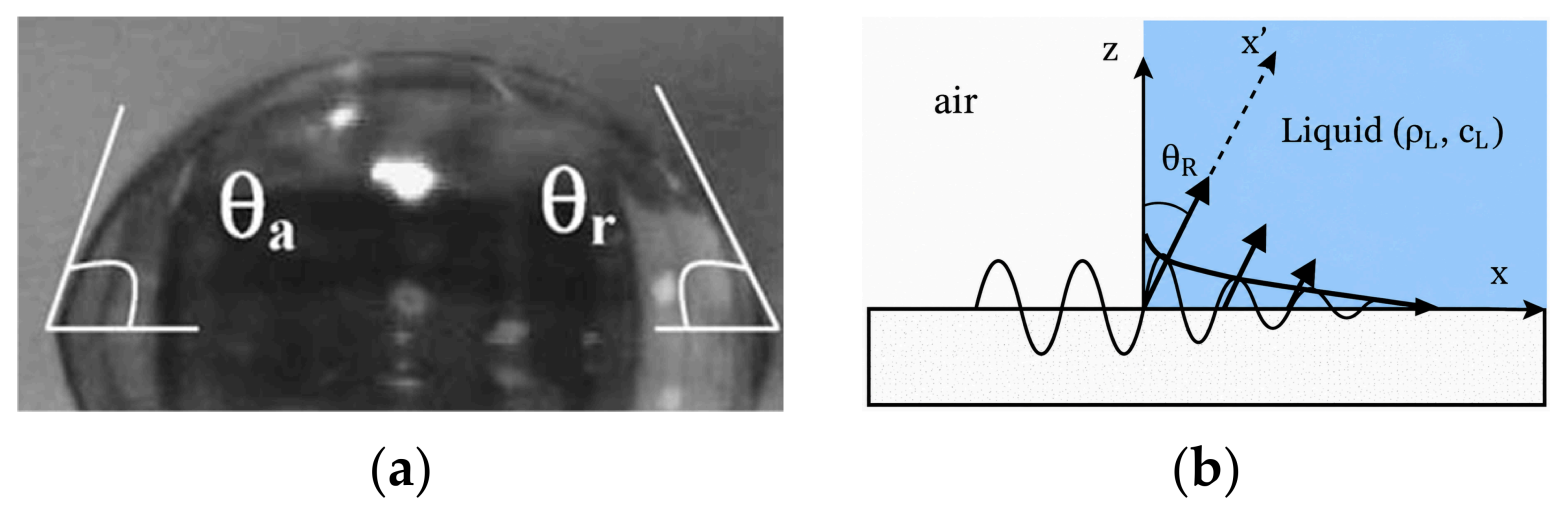
Image (**a**) shows the droplet symmetry at rest with equal advancing and receding angles [26] and image (**b**) represents the longitudinal wave leaking inside the liquid with a diffraction angle $\theta_{R}$ [22].

To understand the phenomena of droplet displacement, the symmetry of the droplet is discussed in terms of contact angles. At rest, the droplet is symmetric, as seen in Figure 7a, and forms a non-linear medium acting as a pressure rectifier in front of the Rayleigh wave [25]. Once the wave comes into contact with the water droplet, the wave starts to attenuate, leaking a longitudinal wave inside the droplet. This causes an internal flow and generates a pressure gradient confined within the droplet due to the difference between its propagation velocity in water and the piezoelectric substrate. This occurs at a diffraction angle $\theta_{R}$, indicated in Figure 7b, which is typically 22°~25° for water on lithium niobate substrates, and is defined by [26]:

(3) $$\theta_{R}=\arcsin\left(\frac{v_{liquid}}{v_{substrate}}\right)$$

This pressure gradient produces a driving force in the propagation direction of the wave and breaks the left–right symmetry of the droplet. This is described by the contact angle hysteresis ∆θ:

(4) $$∆\theta =\theta_{a}-\theta_{r}$$

The force $F_{S}$ applied on the particles in the droplet is defined by:

(5) $$F_{S}=2R\sigma sin\left(\frac{\theta_{a}-\theta_{r}}{2}\right)(cos\theta_{a}-{cos\theta }_{r})$$

where *R* is the droplet radius, *σ* is the liquid–vapor surface tension, and *θ_a_* and *θ_r_* are the advancing and receding contact angles [24].

As the droplet asymmetry increases with increasing RF power and the internal flow intensifies forming inner vortices, the droplet starts to stretch and to flatten, creating a forward displacement [27]. While the droplet displacement mechanism appears to be simple, both the acoustic streaming effect and the acoustic radiation pressure contribute to this phenomenon. Moreover, several factors, including surface nature, wave type and wavelength, RF power, acoustic displacement, droplet size, and working frequency, affect the droplet motion.

To proceed, the research objectives focused on assessing the effectiveness of the cleaning system, followed by evaluating its effect on the measurements’ repeatability after cleaning. Hence, the experimental work was divided into several experiments, to each its specific purpose.

### 2.3. Test Bench Configuration

To evaluate the new SAW chip, an experimental setup consisting of an aerosol generation bench and dedicated electronics for SAW sensors was developed.

#### 2.3.1. Aerosol Generation

An experimental setup was designed to evaluate the performance of the sensors in the cascade impactor by recording particle measurement. It consists of a controlled 1 m^3^ chamber, in which the sensor-based cascade impactor is placed. An aerosol generator, PALAS AGK 2000^®^ (PALAS GmbH, Karlsruhe, Germany), is also placed inside the chamber. It uses suspension solutions to generate aerosols by atomization in a repeatable manner, with long-term dosing and in a size range that can go from 5 nm to 15 µm. A LabVIEW interactive interface is also available to control the pre-pressure given to the generator by means of a pressure controller. The cascade impactor is connected to a Gilian^®^ pump of type Gilair™ Plus (SENSIDYNE Manufacturer, St. Petersburg, FL, USA) that serves to collect aerosol samples through the impactor. For the purposes of repeatability and comparison, the inside of the chamber is monitored using the Fidas^®^ OPC from PALAS^®^ (PALAS GmbH, Karlsruhe, Germany). It measures the concentrations of particles (PM1, PM2.5, PM4, PM10 and PMtot) and serves as a reference.

#### 2.3.2. Electronics

**Figure 8:**
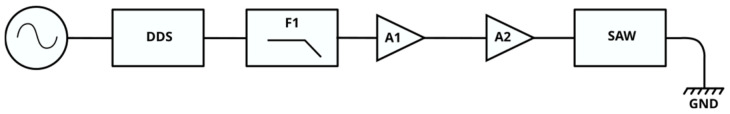
Block diagram of the bench built to induce droplet displacement via SAWs for surface cleaning, where DDS is a Direct Digital Synthesizer, F1 represents a filter, A1 and A2 are power amplifiers, and GND denotes ground.

The electronics used for the sensor-cleaning application were specially built and developed for this purpose and were initially presented in the work of Paquit [18]. The sensors at each stage are interrogated using electronics developed in our laboratory by Rabus [28]. It provides the sensors’ transfer function in terms of phase and amplitude and monitors the phase in open-loop mode at constant frequency. For the cleaning experimental bench, a sinusoidal signal and a frequency synthesizer are used to control the device’s central frequency. A series of RF power amplifiers are added to amplify the signal. RF signals with various powers are transmitted to the electrodes at the resonant frequency of the transducer and are responsible for the displacement of the water droplet deposited on the sensor surface. At each RF power, a droplet behavior analysis was conducted. As for the resonant frequency, it was determined by measuring the minimum of the S_11_ scattering parameter of each of our devices. The setup is schematized in Figure 8. The droplet displacement was then monitored using the KEYENCE VHX-7000 digital microscope (KEYENCE France SAS, Bois-Colombes, France).

## 3. Results

Besides the design of the new SAW chip, two key parameters had to be determined: the droplet size and the RF power. Wave characterization was first performed to determine the wave amplitude. This was followed by a series of experiments aimed at identifying the optimal values of the aforementioned parameters. Subsequently, experiments were conducted to demonstrate the feasibility of the SAW-induced droplet cleaning system and to evaluate its influence on the sensor measurements. 

### 3.1. Wave Characterization Using the Laser Heterodyne Interferometer

**Figure 9:**
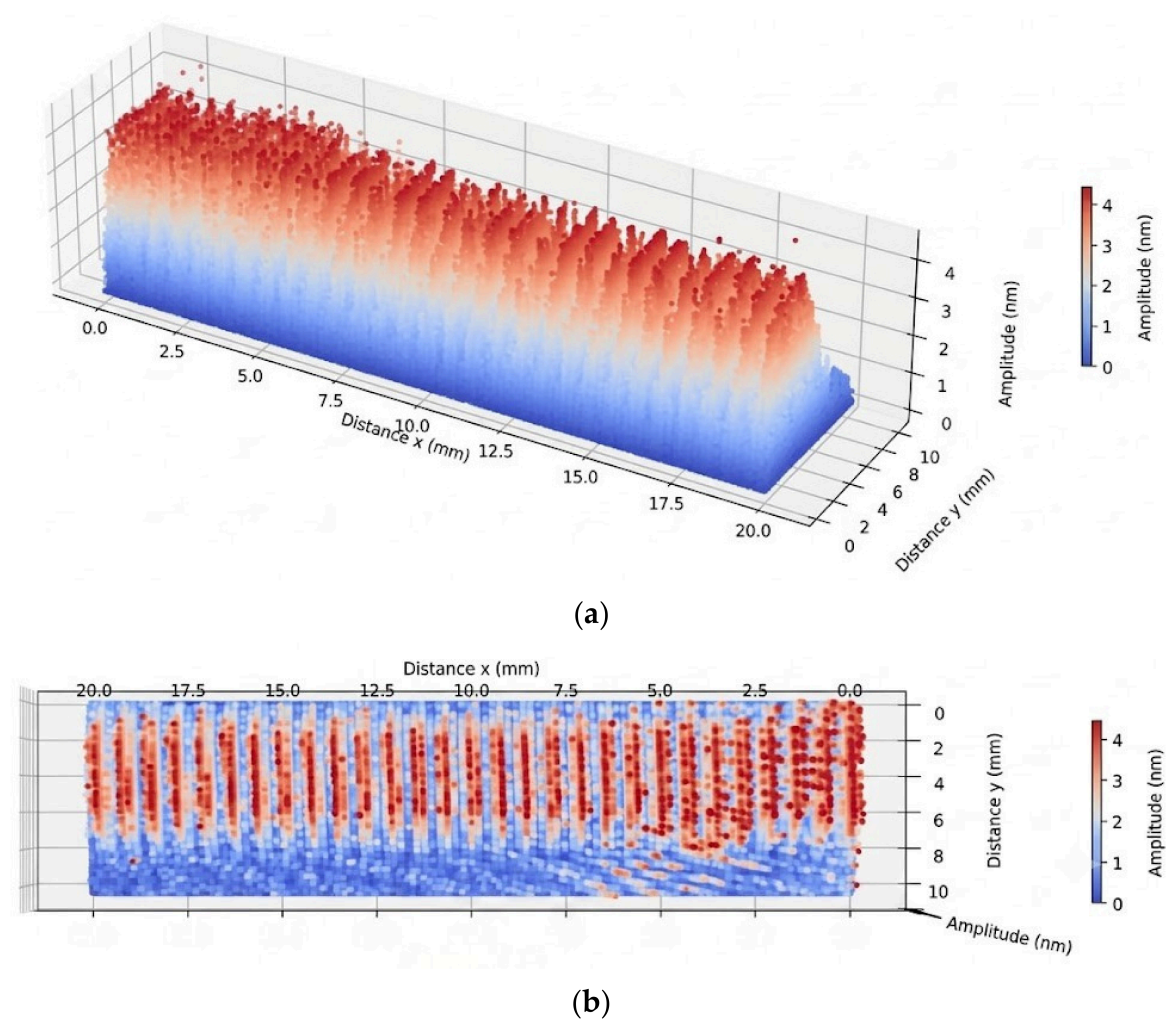
The wave characterization done using a laser heterodyne interferometer showing in (**a**) the wave’s amplitude of 4 nm and in (**b**) its propagation width, which corresponds to the acoustic aperture of the considered sensor.

As detailed in the previous sections, our original delay-line sensor based on lithium niobate excites Rayleigh waves at 98 MHz. The innovative design consisted of integrating an additional IDT dedicated to surface cleaning, positioned alongside the existing structure and generating Rayleigh waves. The out-of-plane component of this latter feature is essential in ensuring the displacement of the droplet. A sufficient acoustic displacement is required, i.e., in the order of a few nanometers. For this purpose, a laser heterodyne interferometer was used to measure the normal displacement of the wave on the substrate surface. The results obtained are shown in Figure 9. The 3D representation of the wave in Figure 9a shows an amplitude of 4 nm. This value is coherent with those reported in the literature capable of displacing a droplet. Figure 9b gives the wave width, which matches the device’s acoustic aperture, which the droplet size should respect.

### 3.2. Preliminary Experiments to Determine the Droplet Size and the Required RF Power

After verifying that the amplitude of the Rayleigh waves is sufficient for particle displacement, a further series of experiments was conducted to determine the water droplet size and the appropriate RF power needed to induce its motion. For confidentiality reasons, only the ranges of droplet sizes and corresponding RF power levels are disclosed. Therefore, droplets with volumes ranging from 1 to 8 µL were tested under RF power levels between 26 and 35 dBm. Starting with small droplets and under low input power, pure droplet vibrations were observed while the droplet remained in place. This behavior persisted even after attempting to increase the input power, and no advancing motion was detected. We then experimented with larger droplets; while keeping the same initial RF power, inner circulations began to appear inside the droplet, highlighting the acoustic streaming effect, and a slight forward deformation became visible. At this point, the RF power was further increased, and the droplet was observed to lose its symmetry and started moving. The experiments revealed that, for a constant droplet size, increasing the RF power resulted in a higher droplet velocity. Conversely, for a constant RF power, smaller droplets exhibited higher velocities than larger ones.

### 3.3. Experimental Validation of Surface Cleaning via SAW-Induced Droplet Displacement

**Figure 10:**
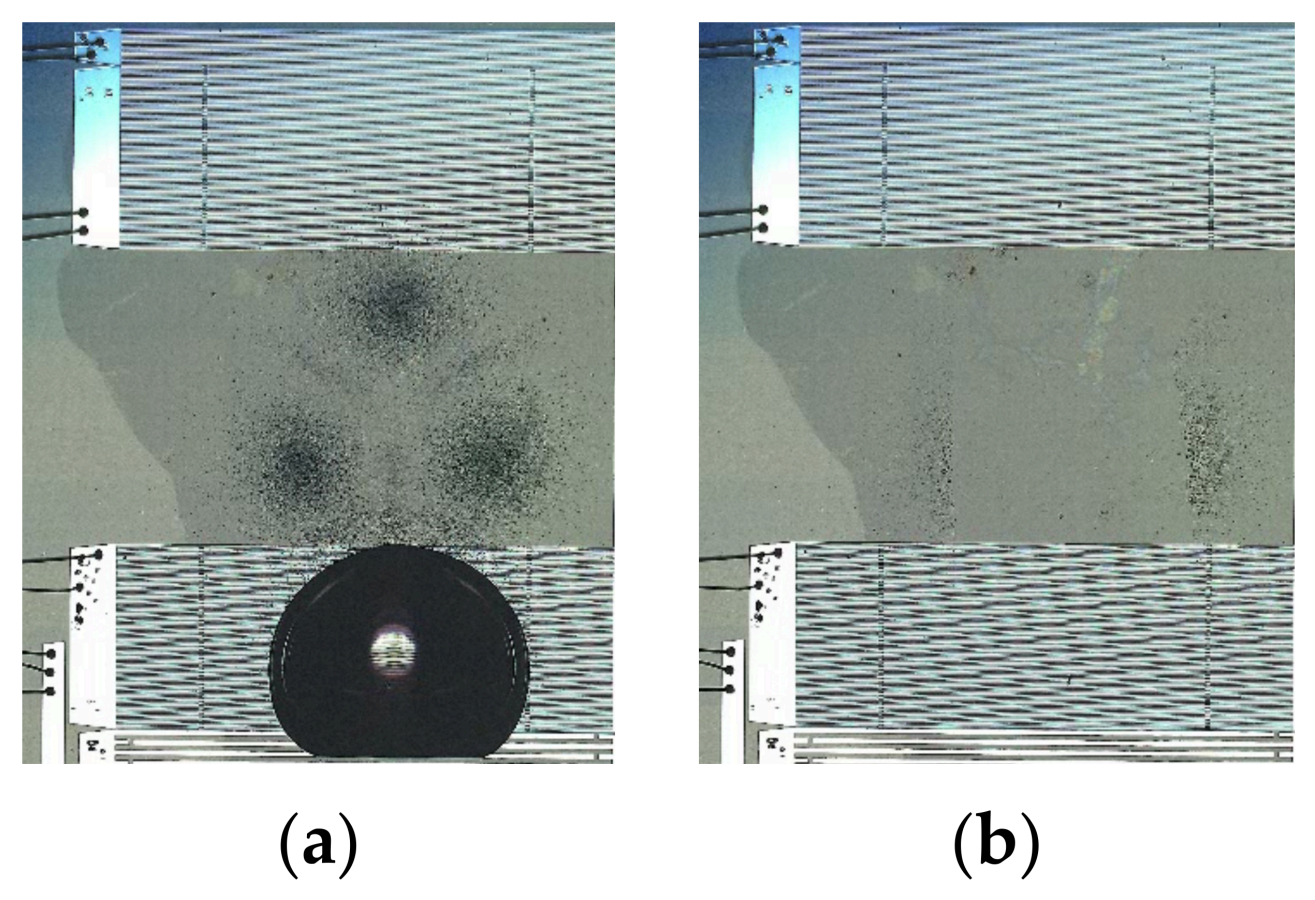
Optical images showing in (**a**) the sensor’s surface impacted with NaCl particles before cleaning and the droplet initially placed on the sensor to displace these particles via SAWs and in (**b**) the sensor’s surface after the cleaning cycle.

After identifying the suitable ranges of droplet size and RF power, subsequent experiments focused on assessing the droplet’s ability to remove impacted particles. Given that the sensing surface has a dimension of approximately 4 mm, large droplets were not considered, as their size exceeds the width of the active region. Several sensors used inside the impactor were considered and exposed to NaCl particles, generating PM2.5*. Optical images were taken using the numerical microscope to monitor the evolution of this cleaning step [12]. In Figure 10a, PM2.5* was collected on the sensing zone after a several-minute sampling period, and the particle coverage fraction was determined to be 22.03 ± 1.10% of the total sensing zone. A droplet matching the dimensions of the active zone was placed on the measurement emitting IDT. For low RF powers, no droplet displacement was observed. The droplet vibrated in place. Intuitively, this was caused by the fact that the cleaning IDT imposed additional surface tension on the droplet. The RF power was then increased, and the droplet began to move slowly, overcoming the electrodes and reaching the active zone. Once in contact with the particles, the droplet entrained them along the surface, crossing the receiving IDT, in approximately 3 s. The particles were transported outside the sensor surface, reaching the PCB, and the droplet evaporated a few seconds after leaving the SAW chip. The region along the droplet trajectory was left clean, with a residual surface coverage of 2.27 ± 0.11%, as seen in Figure 10b. Droplet evaporation is not a concern in our application, as the droplet traveled a distance of 4 mm within a few seconds to reach the end of the active zone, and an additional 3 mm to reach the edge of the chip where the particles are evacuated.

Having experimented with fluid particles such as NaCl, it was essential to repeat the assessment using solid particles to validate the feasibility of using SAW-actuated droplets for their removal. Experiments with SiC particles showed results similar to those obtained with NaCl solutions.

### 3.4. Measurement Results: Assessment of the Impact of the Cleaning Mechanism on Sensor Measurements

After demonstrating the feasibility of using SAWs to displace a droplet for surface cleaning, it was necessary to assess the impact of the cleaning process on sensor measurements. The objective was to evaluate the repeatability of the measurements; therefore, a series of experiments was conducted. The SAW chip was mounted at the PM2.5* stage throughout the entire experiment; thus, the same sensor was used for the entire evaluation. The experiments consisted of collecting particles on the sensor inside the impactor, followed by a cleaning cycle. A solution of NaCl was prepared and set to generate 120 ± 6 µg/m^3^ of PM2.5*. A first sampling was performed to contaminate the surface with particles, followed by a cleaning cycle to initiate the procedure. This was followed by four cycles of particle measurement and particle cleaning steps. Every measurement cycle comprised two consecutive sampling and measurement steps. The results are logged in Table 1, where the PSVs are reported. To recall, these values are extracted from the direct response of the sensor, giving the variation in phase over time, at a constant PM concentration and a constant frequency.

## 4. Discussion

The preliminary experiments were essential in determining the optimal droplet size and RF input power for SAWs to displace the droplet using the adopted cleaning IDT design. Droplets smaller than 2 µL were disregarded as they only exhibited internal circulations rather than displacement. Droplets larger than the sensor surface were also excluded. Moreover, the driving force responsible for the displacement was proven to be directly proportional to the RF input power. This means that higher RF power results in faster displacement. However, very high RF powers are not recommended to avoid the risk of damaging the electrodes. Following these tests, the optimal droplet size and the suitable RF power were determined to displace the droplet using the innovative design of the chip and the cleaning IDT. Further investigation was then carried out to assess the ability of the droplet to carry particles outside the sensing zone. Experiments using PM2.5* NaCl particles and PM10* SiC particles were conducted. In either case, the droplet in the abovementioned conditions was able to carry the particles while moving, irrespective of their nature and size. The acoustic wave induced vortices within the droplet, which were seen under the microscope in real time. The sensor surface before and after applying the cleaning cycle was characterized using a numerical microscope, and the particle coverage areas were quantified. This assessment confirmed that the exploitation of SAWs with the intermediary of a water droplet can be used for surface cleaning, as the images taken following the droplet displacement revealed a clean sensing zone with a significantly reduced particle coverage area with respect to the collection surface.

After the optical validation of the effectiveness of this technique in restoring the initial surface conditions, we verified whether the cleaning process affected the sensor’s measurements. This was done by recording the PSV during several cleaning and measurement cycles, as reported in Table 1. Comparing the first measurements following all four cleaning cycles, the PSV values indicated good repeatability, with a percentage error of approximately 1.20%. This confirms that successive cleaning cycles did not affect the sensor’s performance. Focusing on the second measurements, particles were sampled again after the first collection, when they had already accumulated on the sensor surface. A higher percentage error of 3.28% was observed, relative to that obtained under the initial conditions. Nevertheless, the readings can still be regarded as repeatable. Comparing the first and second measurements across all samplings, the second values were consistently lower than the corresponding initial readings. This indicates a damping effect, with an error of approximately 23%. This behavior was expected given the experimental conditions, in which a high particle concentration (120 µg/m^3^) was generated. This highlights the effect of surface saturation, in a highly contaminated environment, on sensor sensitivity and reinforces the need for a self-cleaning mechanism. Overall, these results confirm that the SAW-actuated water droplet can restore the initial surface condition of the sensor without degrading its performance, and that the dual functionality of the new SAW chip is validated. The PSV values recorded after several cleaning cycles remain repeatable, supporting this conclusion.

## 5. Conclusions

In this article, we presented an original SAW chip dedicated to particle measurement in real time, as well as surface cleaning. The sensors are used as part of a prototype based on a four-stage cascade impactor to measure particles of different sizes. The system functions properly; however, over extended periods of use, a limitation was identified, that is, surface saturation of the sensors, which degrades their sensitivity. To upgrade our system and extend its durability, we had to address the problem of the sensors’ maintenance by equipping the system with a self-regenerating mechanism. We proposed an elegant solution to resolve this issue by exploiting SAWs, forming the basis of this study. The cleaning mechanism uses these waves to displace a water droplet under a specific RF power and remove the particles from the collection surface. This is possible due to the interplay of two phenomena: the acoustic streaming effect and the acoustic radiation pressure. In this paper, we presented our innovative SAW chip comprising, on the same piezoelectric substrate, a double-delay line for particle measurement and an IDT for surface cleaning. We then proceeded to determine the parameters governing this application, namely the water droplet size and the RF power. Through extensive experimentation, we identified the optimal droplet size for this purpose, consistent with the dimensions of our sensor. Furthermore, the power needed to actuate this droplet was also determined. An optical validation was conducted using NaCl and SiC particles and showed that the droplet was able to displace the particles from the sensor surface. Additional experiments were also carried out to evaluate the effect of this technique on the sensor’s sensitivity. The goal was to evaluate the repeatability of measurements after several cleaning cycles. The results were encouraging and the measurements were proven to be repeatable, indicating a negligible influence of the cleaning process. Although this study yielded several positive outcomes, the autonomous functionality of the mechanism is still under development to enable the droplet to reach the surface when needed without human intervention. The durability and aging of IDTs are also being investigated, especially since we are using RF power to induce droplet displacement. Once these are finalized, we will have a complete autonomous system capable of performing real-time particle separation and measurement, while incorporating an autonomous self-cleaning mechanism responsible for ensuring durability and preventing sensor surface fouling. As no single device currently incorporates all of these characteristics simultaneously, these advancements represent a significant step forward in the field.

## 6. Patents

The research conducted in this work resulted in a patent that is in the process of validation. It also received recognition in the IEEE IFCS-EFTF joint symposium held in Mexico in 2025, where it was awarded the Best Poster Prize.

## Figures and Tables

**Table 1:** PSV measured after a series of cleaning cycles, followed by two 5-min successive samplings at 3 Lpm. A PM2.5* concentration of 120 µg/m^3^ was considered throughout the entire experiment.

Measurement Condition	Phase Shift Velocity, PSV (°/s)	
	1st Measurement	2nd Measurement
Measurement after the first cleaning	−2.705 × 10^−2^	−2.190 × 10^−2^
Measurement after the second cleaning	−2.788 × 10^−2^	−2.223 × 10^−2^
Measurement after the third cleaning	−2.808 × 10^−2^	−2.018 × 10^−2^
Measurement after the fourth cleaning	−2.758 × 10^−2^	−2.111 × 10^−2^
Percentage error	1.20%	3.28%

## Data Availability

Research data are available upon request, due to confidentiality.

## Abbreviations

The following abbreviations are used in this manuscript:

IDTs	InterDigital Transducers (or InterDigitated Transducers)
OPC	Optical Particle Counter
PCB	Printed Circuit Board
PM	Particulate Matter
PSV	Phase Shift Velocity
RF	Radio-Frequency
SAW	Surface Acoustic Wave

## References

[1] Grimm Grimm Portable Environmental Dust Monitor-11-E. S&V Samford Instruments Ltd. https://sv.svsamford.com/air-quality-monitors-multi-gas-monitor-iaw-monitor-aerosol-measuring-solutions/47-grimm-aerosol-environmental-dust-monitor-mini-laser-aerosol-spectrometer.html.

[2] Honeywell C7363 Particulate Matter Sensor. https://buildings.honeywell.com/us/en/products/by-category/sensors/particle-counters/c7363-particulate-matter-sensor.

[3] Thermo Scientific 1405 TEOM^TM^ Continuous Ambient Particulate Monitor. https://www.thermofisher.com/order/catalog/product/TEOM1405.

[4] Dekati PM10 Impactor. https://dekati.com/products/dekati-pm10-impactor/.

[5] California Measurements, Inc Model PC-2 -Real-Time Air Particle Analyzer. https://www.environmental-expert.com/products/pc-2-real-time-air-particle-analyzer-787557.

[6] TSI Incorporated Quartz Crystal Microbalance QCM MOUDI^TM^ Impactor 140. https://tsi.com/products/cascade-impactors/real-time-qcm-moudi/quartz-crystal-microbalance-qcm-moudi-impactor-140/.

[7] Chen M., Romay F.J., Li L., Naqwi A., Marple V.A. (2016). A novel quartz crystal cascade impactor for real-time aerosol mass distribution measurement. Aerosol Sci. Technol..

[8] Djoumi L. (2017). Developpement de Capteurs à Ondes Elastiques de Surface Pour la Mesure de la Concentration des Particules Fines dans l’Air. Ph.D. Dissertation.

[9] Djoumi L., Vanotti M., Blondeau-Patissier V. (2018). Real Time Cascade Impactor Based on Surface Acoustic Wave Delay Lines for PM10 and PM2.5 Mass Concentration Measurement. Sensors.

[10] Fawaz G. (2025). Optimization of a Cascade Impactor Equipped with Self-Cleaning SAW Sensors for Particle Measurement: Development and Design, Experimental Validation and Efforts Towards the Use of POI Substrates. Ph.D. Dissertation.

[11] Fawaz G., Poisson S., Vanotti M., Soumann V., Baron T., Blondeau-Patissier V. Surface Cleaning of Microparticles Sensors Integrated in a Cascade Impactor via Water Droplet Displacement Using Surface Acoustic Waves. Proceedings of the 2025 Joint Conference of the European Frequency and Time Forum and IEEE International Frequency Control Symposium (EFTF/IFCS).

[12] Sauerbrey G. (1959). Verwendung von Schwingquarzen zur Wägung dünner Schichten und zur Mikrowägung. Z. Phys..

[13] Kurosawa M., Watanabe T., Futami A., Higuchi T. (1995). Surface acoustic wave atomizer. Sens. Actuators A Phys..

[14] Renaudin A., Tabourier P., Zhang V., Druon C., Camart J.-C. (2006). Plateforme SAW dédiée à la microfluidique discrète pour applications biologiques. Houille Blanche.

[15] Wixforth A. (2003). Acoustically driven planar microfluidics. Superlattices Microstruct..

[16] Alzuaga S., Daniau W., Manceau J.F., Ballandras S., Bastien F. Displacement of droplets on a surface using ultrasonic vibration. Proceedings of the World Congress on Ultrasonics: WCU 2003.

[17] Brunet P., Baudoin M., Matar O.B., Zoueshtiagh F. (2010). Droplets displacement and oscillations induced by ultrasonic surface acoustic waves: A quantitative study. Phys. Rev. E.

[18] Paquit M., Djoumi L., Vanotti M., Soumann V., Martin G., Blondeau-Patissier V. Displacement of Microparticles on Surface Acoustic Wave Delay Line Using High RF Power. Proceedings of the 2018 IEEE International Ultrasonics Symposium (IUS).

[19] Alzuaga S. (2004). Manipulation de Gouttes de Liquides par des Méthodes Acoustiques. Ph.D. Dissertation.

[20] Song H., Lee J., Lee K.Y., Chung S.K. Self-Cleaning Device Using SAW Actuation. Proceedings of the 2020 IEEE 33rd International Conference on Micro Electro Mechanical Systems (MEMS).

[21] Renaudin A., Tabourier P., Zhang V., Camart J.C., Druon C. (2006). SAW nanopump for handling droplets in view of biological applications. Sens. Actuators B Chem..

[22] Bennes J., Alzuaga S., Ballandras S., Cherioux F., Bastien F., Manceau J.-F. Droplet ejector using surface acoustic waves. Proceedings of the IEEE Ultrasonics Symposium.

[23] Qi A., Friend J.R., Yeo L.Y. Investigation of SAW atomization. Proceedings of the 2009 IEEE International Ultrasonics Symposium.

[24] Daru V., Reyt I., Bailliet H., Weisman C., Baltean-Carlès D. (2017). Acoustic and streaming velocity components in a resonant waveguide at high acoustic levels. J. Acoust. Soc. Am..

[25] Baudoin M., Brunet P., Matar O.B., Herth E. (2012). Low power sessile droplets actuation via modulated surface acoustic waves. Appl. Phys. Lett..

[26] Beyssen D., Le Brizoual L., Elmazria O., Alnot P. (2006). Microfluidic device based on surface acoustic wave. Sens. Actuators B Chem..

[27] Riaud A., Baudoin M., Matar O.B., Thomas J.-L., Brunet P. (2017). On the influence of viscosity and caustics on acoustic streaming in sessile droplets: An experimental and a numerical study with a cost-effective method. J. Fluid Mech..

[28] Rabus D. (2013). Resonateur à Ondes Élastiques de Volume à Harmoniques Élevés (HBARs) Pour Mesures Gravimetriques: Application à la Détection de Gaz. Ph.D. Dissertation.

